# Inflammatory pseudotumor: big similarities and diagnostic challenges

**DOI:** 10.1093/jscr/rjae562

**Published:** 2024-09-04

**Authors:** Rihane El Mohtarim, Sabrine Derqaoui, Samia Sassi, Omar Belkouchi, Amina El Alaoui Babana, Khaoula Boumeriem, Kaoutar Imrani, Ittimade Nassar, Ahmed Jahid, Fouad Zouaidia, Kaoutar Znati, Abdelkader Belkouchi, Zakia Bernoussi

**Affiliations:** Department of Pathology, Ibn Sina Teaching Hospital, Abderrahim Bouabid Avenue, Rabat 12000, Morocco; Mohammed V University, Nations Unies Avenue, Agdal, Rabat 12000, Morocco; Department of Pathology, Ibn Sina Teaching Hospital, Abderrahim Bouabid Avenue, Rabat 12000, Morocco; Mohammed V University, Nations Unies Avenue, Agdal, Rabat 12000, Morocco; Department of Pathology, Ibn Sina Teaching Hospital, Abderrahim Bouabid Avenue, Rabat 12000, Morocco; Mohammed V University, Nations Unies Avenue, Agdal, Rabat 12000, Morocco; Mohammed V University, Nations Unies Avenue, Agdal, Rabat 12000, Morocco; Department of General Surgery “A”, Ibn Sina Teaching Hospital, Abderrahim Bouabid Avenue, Rabat 12000, Morocco; Mohammed V University, Nations Unies Avenue, Agdal, Rabat 12000, Morocco; Department of General Surgery “A”, Ibn Sina Teaching Hospital, Abderrahim Bouabid Avenue, Rabat 12000, Morocco; Mohammed V University, Nations Unies Avenue, Agdal, Rabat 12000, Morocco; Department of Radiology, Ibn Sina Hospital University Center, Ibn Sina Teaching Hospital, Abderrahim Bouabid Avenue, Rabat 12000, Morocco; Mohammed V University, Nations Unies Avenue, Agdal, Rabat 12000, Morocco; Department of Radiology, Ibn Sina Hospital University Center, Ibn Sina Teaching Hospital, Abderrahim Bouabid Avenue, Rabat 12000, Morocco; Mohammed V University, Nations Unies Avenue, Agdal, Rabat 12000, Morocco; Department of Radiology, Ibn Sina Hospital University Center, Ibn Sina Teaching Hospital, Abderrahim Bouabid Avenue, Rabat 12000, Morocco; Department of Pathology, Ibn Sina Teaching Hospital, Abderrahim Bouabid Avenue, Rabat 12000, Morocco; Mohammed V University, Nations Unies Avenue, Agdal, Rabat 12000, Morocco; Department of Pathology, Ibn Sina Teaching Hospital, Abderrahim Bouabid Avenue, Rabat 12000, Morocco; Mohammed V University, Nations Unies Avenue, Agdal, Rabat 12000, Morocco; Department of Pathology, Ibn Sina Teaching Hospital, Abderrahim Bouabid Avenue, Rabat 12000, Morocco; Mohammed V University, Nations Unies Avenue, Agdal, Rabat 12000, Morocco; Mohammed V University, Nations Unies Avenue, Agdal, Rabat 12000, Morocco; Department of General Surgery “A”, Ibn Sina Teaching Hospital, Abderrahim Bouabid Avenue, Rabat 12000, Morocco; Department of Pathology, Ibn Sina Teaching Hospital, Abderrahim Bouabid Avenue, Rabat 12000, Morocco; Mohammed V University, Nations Unies Avenue, Agdal, Rabat 12000, Morocco

**Keywords:** inflammatory pseudotumor, surgery, histopathology, immunohistochemistry, case report

## Abstract

Inflammatory pseudotumors are reactive and rare lesions of unknown origin. They are locally aggressive, but they generally do not extend beyond the affected organ, with a favorable prognosis after radical surgical resection. Symptoms and radiologic features are often nonspecific, and pathological examination plays a pivotal role in the diagnosis of inflammatory pseudotumors along with immunohistochemical staining to rule out differential diagnosis, especially myofibroblastic inflammatory tumor. Molecular biology can play a decisive role when these latter cannot establish a definitive diagnosis. Herein, we present a case study that describes a 65-year-old woman presenting a mesocolic mass. The patient underwent a surgical excision, which included a wedge gastric resection, transverse colectomy, and colocolic side-to-side stapled anastomosis. Histopathology and immunohistochemistry confirmed an inflammatory pseudotumor. Surgery remains the main treatment and depends on the location of the tumor. No recurrences are noted; however, a standardized follow-up protocol for inflammatory pseudotumors has yet to be established.

## Introduction

The term ‘inflammatory pseudotumor’ (IPT) was first described in 1939 to characterize a group of fibroinflammatory conditions, having both reactive and neoplastic origins.

It was long considered with inflammatory myofibroblastic tumor (IMT) as two sides of the same coin. However, IPT, classified as a reactive lesion, does not recur after resection and does not metastasize; on the contrary of IMT, which is considered as an intermediate-grade neoplastic entity, often has a high recurrence rate after excision and shows low metastatic potential. The etiology and pathogenesis remain unclear. IPT is often associated with infections, autoimmune diseases, or a posttraumatic context.

This case report aims to mention the importance of a complete surgical resection for a definitive remedy as well as histopathological examination along with immunohistochemistry to sign the diagnosis of IPT and to rule out the differential diagnosis [1].

## Case report

A previously healthy 65-year-old woman was admitted to the Department of Surgery with acute abdominal pain, emesis, chronic constipation, and significant weight loss. Physical examination was normal as well as CEA and CA19–9 levels.

**Figure 1:**
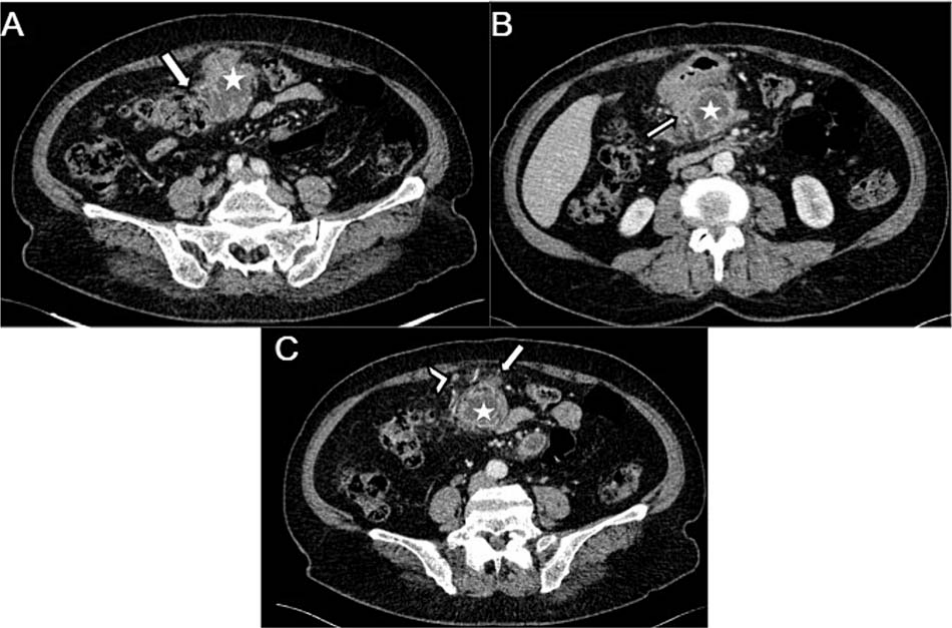
Axial sections of an abdominal CT showing (A) a heterogenous mesocolic mass (white star) infiltrating the transverse colon (White arrow), (B) the pre-pancreatic mass (white star) infiltrating the adjacent gastric wall (white arrow), (C) the mesecolic mass (white star) with stranding of the adjacent fat (white arrow) and satellite lymph nodes (white arrow head).

The abdominal CT scan revealed a pre-pancreatic mass occupying the greater omentum, specifically located in the mesocolon, measuring 54 × 66 × 99 mm. The mass was fairly well-defined, with irregular contours, and had a heterogeneous density. It contained liquid areas in its center and showed peripheral enhancement. This mass infiltrated the transverse colon (Fig. 1A), the head of the pancreas, and the adjacent gastric wall (Fig. 1B) with no vascular invasion. Few satellite lymph nodes were noted (Fig. 1C). Following the imaging results, a GIST or complicated duodenal diverticula was suspected.

The patient was admitted to the operating room and a surgical excision of the mass was performed by laparotomy through a midline supraumbilical incision.

**Figure 2:**
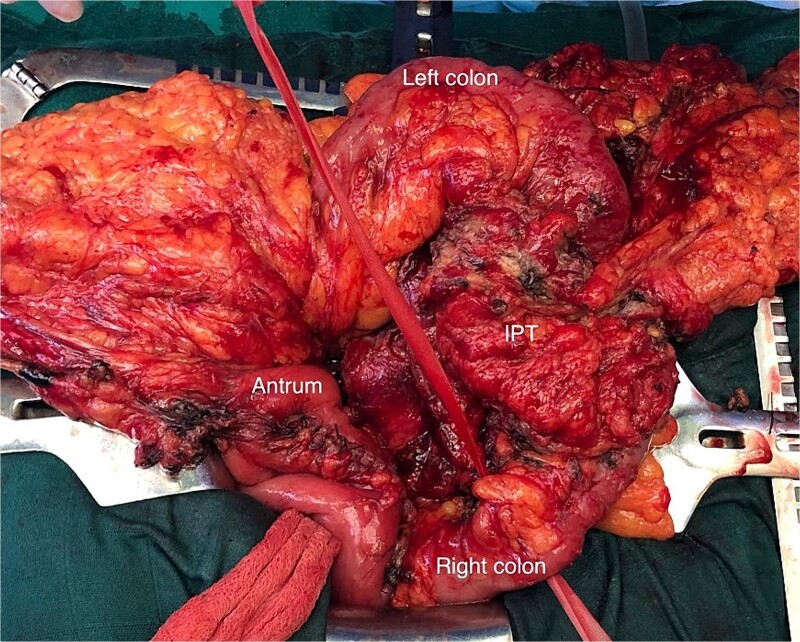
Operative view of the transverse colon tumor (IPT) after freeing the gastric antrum.

Exploration found no metastasis or carcinomatosis, but a locally advanced tumor of the transverse colon with invasion of the gastric antrum, the head of the pancreas, and the root of the mesentery. Dissection was carried out after freeing the pancreas, the duodenum, and the SMV, as the mass was intraoperatively not invading the pancreas (Fig. 2).

**Figure 3:**
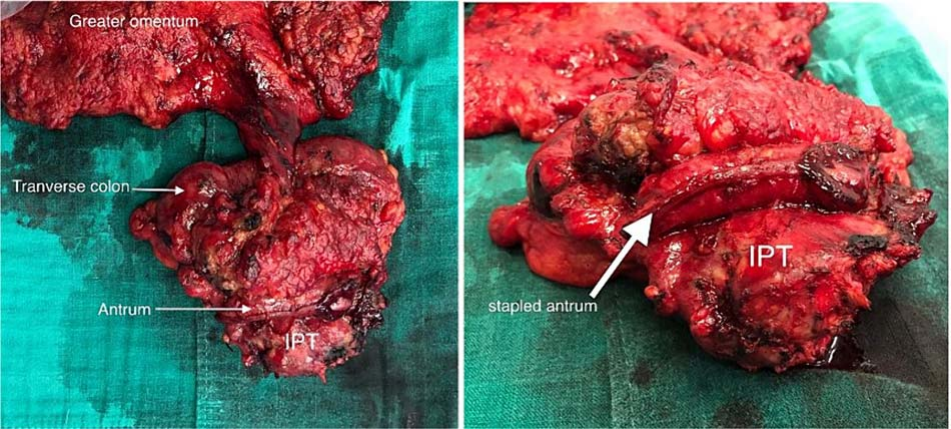
Macroscopic view of the specimen.

A wedge gastric resection was carried on with a transverse colectomy after ligation of the middle colonic pedicle with lymphadenectomy, and a colocolic side-to-side stapled anastomosis was performed (Fig. 3). Drainage was performed at the end of the procedure.

**Figure 4:**
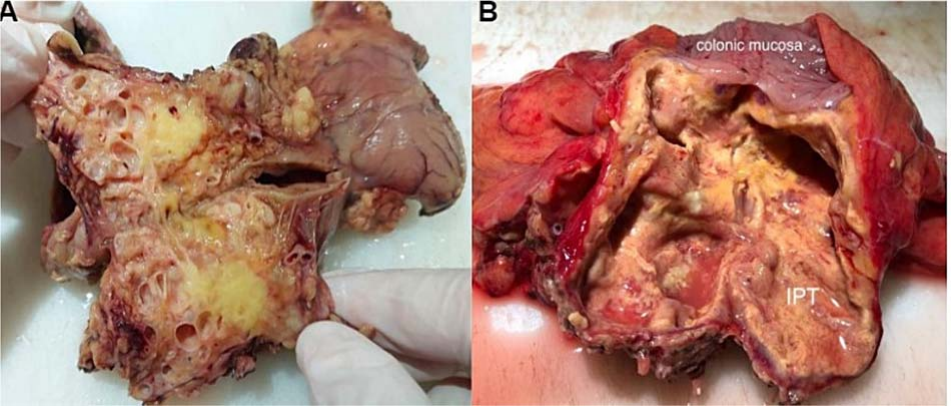
Macroscopic view after section of the specimen showing a cysticized mass with a yellowish appearance (A) and extension to the colonic subserosa (B).

**Figure 5:**
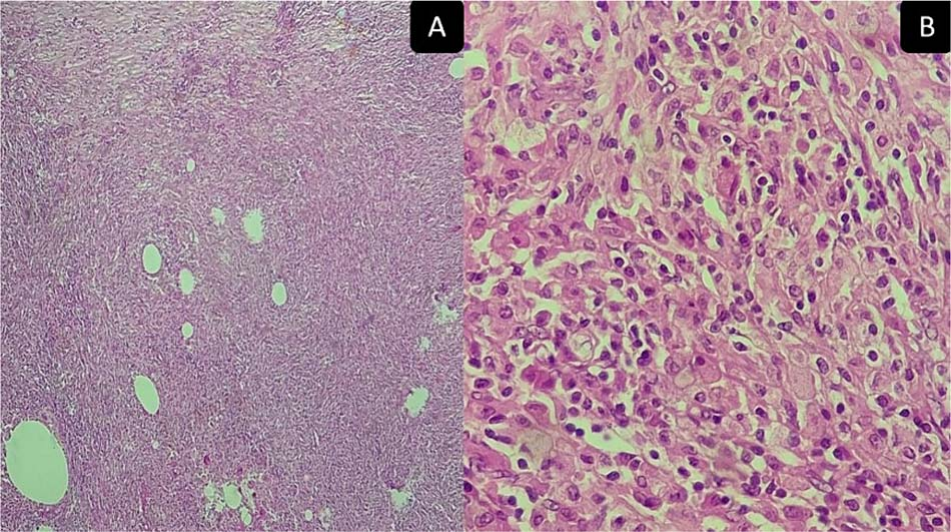
Inflammatory pseudotumor. Histological aspects: desmoid-type fibromatosis conventional pattern histological and immunohistochemical aspects: areas of histiocytes, and myofibroblasts, plasma cells, lymphocytes, and a few neutrophils and eosinophils. (A) Low magnification ×50 and (B) high magnification ×400.

The removed mass was sent to our department for histopathological study. On gross, the mesocolic mass was received with a colonic resection measuring 12 cm, associated with a gastric segment measuring 6 × 2 cm and including the omentum measuring 20 cm. Section showed a cysticized, yellowish mass measuring 10 × 6 × 4 cm, located 3 and 2 cm from the colonic limits and 3.5 cm from the gastric limit (Fig. 4A). The tumor reaches the excision limits and extends to the gastric and colonic subserosa (Fig. 4B). Histologically, the lesion was made up of areas of histiocytes, alternating between foamy and spindle-shaped, associated with numerous myofibroblasts, plasma cells, lymphocytes, and a few neutrophils and eosinophils (Fig. 5A and B). No suspicious cells were seen. The lesion extended to the colonic and gastric subserosa. A tiny liver parenchyma was incidentally discovered on a microscope showing non-specific chronic fibro-inflammatory changes. Pancreatic tissue was additionally noted and was free from specific lesions or tumors. Reactive lymphadenitis was noted.

**Figure 6:**
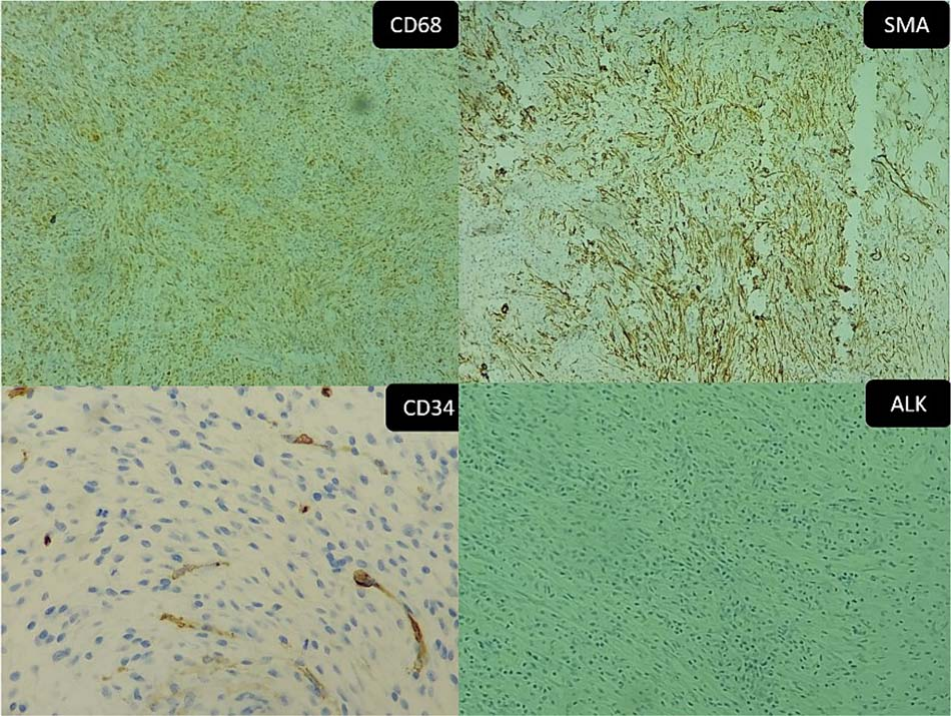
Inflammatory pseudotumor. Immunohistochemical aspects: (A) positive CD68 (original magnification ×50), (B) positive SMA (original magnification ×100), (C) negative CD34 (Original magnification ×200), and (D) negative ALK (original magnification ×50).

The immunohistochemical study showed positivity of myofibroblasts for SML and positivity of histiocytes for CD68, while ALK and AE1AE3 were negative. All those findings were consistent with IPT (Fig. 6A–D).

The postoperative course was uneventful, and the patient didn’t develop any fistula.

## Discussion

IPTs and IMT were long considered as a unique entity, and all data collected from reputed sources, such as Scopus, PubMed, or Google Scholar, show no difference between them [2].

IPT designates all reactive mass-forming lesions that can occur anywhere in the body. They don’t recur after resection, and they don’t metastasize, unlike IMT, which is considered as an intermediate-grade neoplastic entity that often has a high recurrence rate after excision and shows low metastatic potential. IPT affects adults and the elderly, with a male predominance [3]. The majority of IPT cases are asymptomatic, and clinical signs, although nonspecific, depend on the location.

The tumor’s etiology can be infectious, involving pathogens, such as Epstein–Barr virus (EBV) and human herpesvirus 8 (HHV-8), or autoimmune, associated with conditions like IgG4-related disease or Sjögren’s syndrome [4].

Radiologic features are not specific due to fibrotic changes, and there are no sources distinguishing imaging characteristics between IPTs and IMTs. On ultrasound, lesions may appear hypoechoic or hyperechoic with either poorly defined or well-defined borders. CT scans also reveal varied appearances, and the lesions may be isodense, hypodense, or hyperdense compared to surrounding tissue. On MR images, IPTs typically demonstrate low signal intensity on both T1- and T2-weighted sequences, indicative of their fibrotic nature. Contrast-enhanced CT and MRI may reveal homogeneous or heterogeneous lesions [5].

Gross features show generally a well-circumscribed mass. The size ranges from 1 to 20 cm [6]. Histologically, IPT shows a population made of fibroblasts and/or myofibroblasts, lymphocytes, plasma cells, eosinophils, and histiocytes [7]. The lymphoplasmacytic infiltrate is more pronounced in IPT compared to IMT [4].

In immunohistochemistry, IPTs show positivity of myofibroblastic population for SMA and positivity of histiocytes for CD68. This tumor lacks expression of ALK-1 antigens, which is a helpful characteristic to distinguish IPTs from IMTs [8].

On the genetic level, the myofibroblastic cells in these lesions do not exhibit genetic alterations or clonality, unlike IMT, making it a reactive lesion rather than a neoplastic one [8].

Surgical resection with negative margins remains the gold standard to treat IPTs and is both therapeutic and diagnostic.

The prognosis of IPTs is excellent, as recurrences and metastasis do not occur.

Differential diagnosis includes on the first level myofibroblastic inflammatory tumor, then inflammatory fibroid polyp, inflammatory well-differentiated or dedifferentiated liposarcoma, nodular fasciitis, gastrointestinal stromal tumor (GIST), and desmoid fibromatosis [6].

Histopathological examination and immunohistochemistry play a pivotal role in the diagnosis of this rare entity. Genetic studies can play a decisive role to rule out ALK rearrangement found in IMTs.

## Conclusion

IPT is a reactive inflammatory forming mass that has an excellent prognosis after complete surgical resection with no recurrences or metastases. To date, there are no sufficient studies about this rare entity in order to standardize the management across all aspects.
